# The multiple indicator multiple cause model for cognitive neuroscience: An analytic tool which emphasizes the behavior in brain–behavior relationships

**DOI:** 10.3389/fpsyg.2022.943613

**Published:** 2022-08-04

**Authors:** Adon F. G. Rosen, Emma Auger, Nicholas Woodruff, Alice Mado Proverbio, Hairong Song, Lauren E. Ethridge, David Bard

**Affiliations:** ^1^Department of Psychology, University of Oklahoma, Norman, OK, United States; ^2^Department of Psychology, University of Milan-Bicocca, Milan, Italy; ^3^Department of Pediatrics, University of Oklahoma Health Sciences Center, Oklahoma City, OK, United States

**Keywords:** cognitive neurosciences, structural equation modeling, systems of equations, power, sensitivity

## Abstract

Cognitive neuroscience has inspired a number of methodological advances to extract the highest signal-to-noise ratio from neuroimaging data. Popular techniques used to summarize behavioral data include sum-scores and item response theory (IRT). While these techniques can be useful when applied appropriately, item dimensionality and the quality of information are often left unexplored allowing poor performing items to be included in an itemset. The purpose of this study is to highlight how the application of two-stage approaches introduces parameter bias, differential item functioning (DIF) can manifest in cognitive neuroscience data and how techniques such as the multiple indicator multiple cause (MIMIC) model can identify and remove items with DIF and model these data with greater sensitivity for brain–behavior relationships. This was performed using a simulation and an empirical study. The simulation explores parameter bias across two separate techniques used to summarize behavioral data: sum-scores and IRT and formative relationships with those estimated from a MIMIC model. In an empirical study participants performed an emotional identification task while concurrent electroencephalogram data were acquired across 384 trials. Participants were asked to identify the emotion presented by a static face of a child across four categories: happy, neutral, discomfort, and distress. The primary outcomes of interest were P200 event-related potential (ERP) amplitude and latency within each emotion category. Instances of DIF related to correct emotion identification were explored with respect to an individual’s neurophysiology; specifically an item’s difficulty and discrimination were explored with respect to an individual’s average P200 amplitude and latency using a MIMIC model. The MIMIC model’s sensitivity was then compared to popular two-stage approaches for cognitive performance summary scores, including sum-scores and an IRT model framework and then regressing these onto the ERP characteristics. Here sensitivity refers to the magnitude and significance of coefficients relating the brain to these behavioral outcomes. The first set of analyses displayed instances of DIF within all four emotions which were then removed from all further models. The next set of analyses compared the two-stage approaches with the MIMIC model. Only the MIMIC model identified any significant brain–behavior relationships. Taken together, these results indicate that item performance can be gleaned from subject-specific biomarkers, and that techniques such as the MIMIC model may be useful tools to derive complex item-level brain–behavior relationships.

## Introduction

Obtaining the highest signal-to-noise ratio in neuroimaging data has encouraged rapid methodological development for cognitive neuroscientists. Necessitated by the difficulty inherent to mapping the human brain where a ground truth is inaccessible. In a similar vein the quantification of cognitive traits lacks a ground truth as well. Cognitive neuroscientists typically employ workflows which minimize the influence of confounding variables in neuroimaging data; however, cognitive stimuli do not typically receive the same scrutiny. In one specific dimension of cognition, socio-emotional functioning, solutions to measuring cognition have been multipronged such as ensuring participants are familiar with the testing environment, as well as ensuring an adequate number of behavioral stimuli are obtained (Brooker et al., 2020). The multiple indicator multiple causes (MIMIC) model with itemset purification represents an additional step cognitive neuroscientists can employ to further ensure the highest quality of cognitive data are obtained. The MIMIC model represents a systems of equations approach that combines both causal and measurement modeling. Causal modeling represents the end goal of most scientific endeavors as it applies theory in a testable manner and a strict application (Rodgers, 2010). Measurement models are desirable as an inherent limitation of cognitive assessments is the influence of measurement error (Bollen, 1989b). Through the joint estimation of both a causal and measurement model, the MIMIC model represents a unique analytic tool for cognitive neuroscience as it ensures a more fine-grained assessment of behavior and a more tightly coupled brain–behavior causal model is obtained.

The application of measurement models is not novel for cognitive neuroscience. Examples exist when linking intelligence to brain volume (Gignac and Bates, 2017), interlocked functional relationships across brain regions (Finn et al., 2015), and electroencephalogram characteristics (McKinney and Euler, 2019; Hakim et al., 2021). These studies typically utilize a two-stage approach where the summary metrics of both behavioral data and neural data are created using techniques such as sum-scores, or principal components analysis, and then brain–behavior relationships are identified using a general linear model. One prominent example found within the magnetic resonance imaging literature includes the FSL FEAT software which estimates mass univariate statistics across the entire human brain using a general linear model (Woolrich et al., 2001). While the linear model has been a great success for mapping structural and functional underpinnings of behavior, techniques which jointly model both brain and behavior in a single system have become increasingly powerful for the identification of brain–behavior relationships.

**Figure 1:**
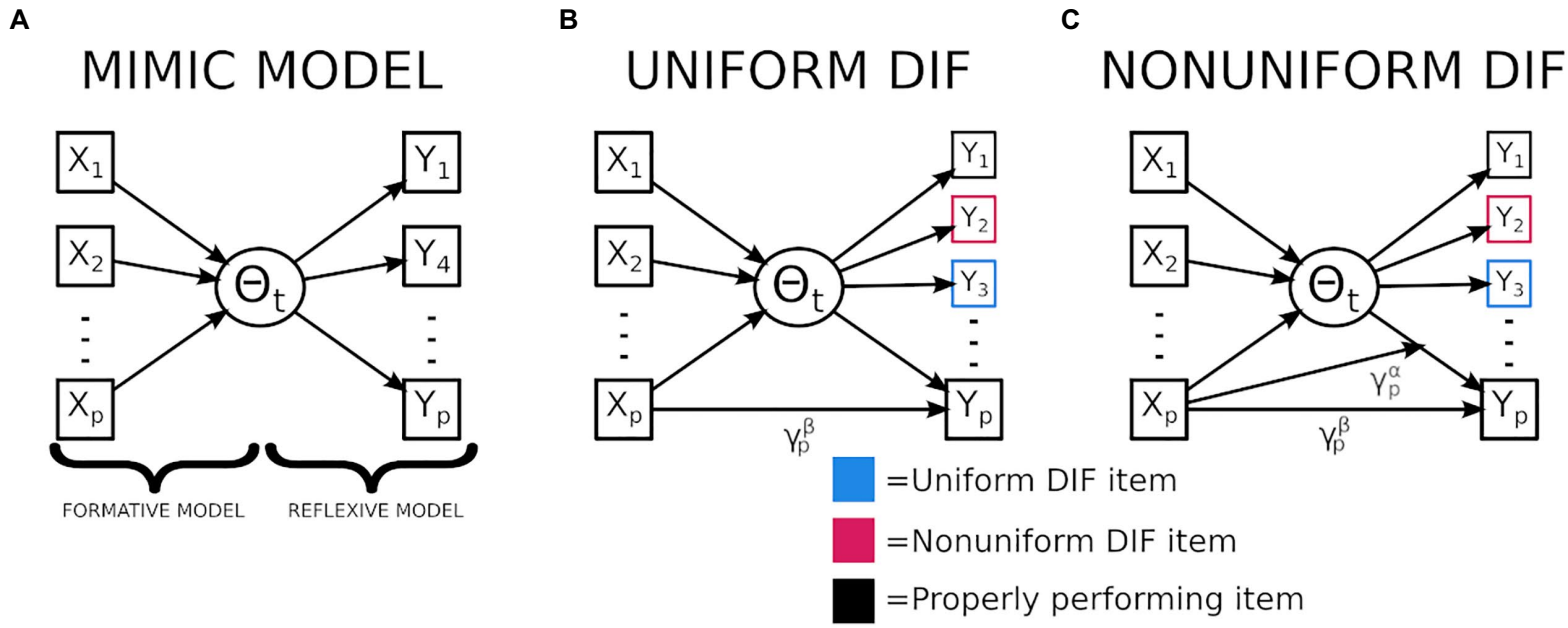
Graphical representation of a multiple indicator multiple cause (MIMIC) model and MIMIC models exploring differential item functioning (DIF). **(A)** Displayed is the MIMIC model which is composed of a formative (causal) and a reflexive (measurement) model. **(B)** Displays the mechanism used to assess for uniform DIF, notably the mediator is the latent variable which is believed to be the mechanism linking the causal and indicator variables. When the gamma path is not fully mediated then uniform DIF is present. **(C)** Displays the mechanisms used to assess nonuniform DIF, notably, when the relationship between the latent variable and an individual indicator varies as a function of the causal variable nonuniform DIF is present.

Examples of techniques used to jointly model brain–behavior relationships include as canonical correlation analysis (CCA; Wang et al., 2020) or partial least squares regression (PLS; Krishnan et al., 2011). These approaches all seek to identify relationships across high dimensional data by performing dimensionality reduction on one or both sets of data and then identify components with the greatest covariance across sets of variables. However both CCA and PLS reflect more exploratory analytic techniques whereas the MIMIC model requires a more confirmatory approach be applied. The confirmatory nature of the MIMIC model requires a set of theorized causal variables (i.e., brain) to be regressed onto a theorized latent trait (i.e., fluid intelligence) which is approximated by an additional set of indicator variables (i.e., behavior; see Figure 1A). Previous research has applied the MIMIC model to explore brain–behavior relationships (Kievit et al., 2011, 2012), allowing researchers to model an individual’s cognitive ability onto their brain volume. Further applications of the MIMIC model within cognitive neuroscience have allowed explorations into whether individual differences are better explained with group factors or continuous covariates (Zadelaar et al., 2019).

In order to underscore the benefits of the MIMIC model the formative and reflexive components are first described in isolation of one another and then the synthesis of these two approaches highlights the benefit of the MIMIC model. The reflexive model’s distinctions will be described using a two-parameter item response theory (IRT) framework (Embretson and Reise, 2000):


$$p_{i}\left(\theta \right)=\frac{1}{1+e^{-a_{i}\left(\Theta -b_{i}\right)}}$$

In the above model $p_{i}\left(\theta \right)$ is the probability of endorsement for an item (typically binary in nature) given an individual’s latent score estimate, $a_{i}$ is the item discrimination, and $b_{i}$ is the item difficulty. The above formula highlights how given a set of manifest variables IRT estimates a probability to endorse a binary item given an items discrimination and difficulty estimates. Greater discrimination values are desirable given their ability to differentiate on ability more precisely, difficulty reflects the location of the probability of endorsement being a 50% chance for a binary item. The above discrimination and difficulty parameters can be used to map out an item’s characteristic curve which is a graphical representation of the amount of information (discrimination) and location (difficulty) of an individual item. When working with binary data the logic of IRT extends beyond the formula to read as:


$$y_{i}^{*}=\left(\begin{array}{cc} 1 & if\;y_{i}^{*}>\gamma_{i},\\ 0 & otherwise \end{array}\right)$$

Where $\tau_{i}$ is a threshold parameter for $y_{i}^{*}$ , and assume that:


$$y_{i}^{*}=\lambda_{i}\eta +\epsilon_{i}$$

Where $\lambda_{i}$ is a loading parameter, and η reflects an individual’s latent ability and $\epsilon_{i}$ reflects the residual variable. The major appeal of reflexive models for cognitive neuroscience is that these models incorporate measurement error, and they allow insights into the quality of the behavioral data in both the dimensionality and the information provided by the indicator variables.

The formative model adheres to the following formulation:


$$\eta =\gamma^{`}x+\zeta$$

Where γ is a vector of the regression coefficients, *x* is a *q* x 1 vector of manifest random variables where *q* is the number of observed variables, and ζ reflects the residual term. This formulation adheres to the underpinnings of most causal models, but more so implies a linear relationship (Muthén, 1985; Pearl, 2012).

The MIMIC model combines these into a system of equations resulting in the following formulation:


$$y_{i}^{*}=\lambda_{i}\left(\gamma^{`}x+\zeta \right)+\epsilon_{i}$$

The important distinction of this approach is the ability to incorporate residual error from both the formative and measurement model, distinguishing the system approach from these models applied in isolation. Further utility of the MIMIC model is the ability to explore the quality and consistency of the indicator variables if additional variables may be influencing the way individuals respond to items which is referred to as differential item functioning (DIF).

The second major benefit of the MIMIC model is the ability to isolate instances of DIF, which exist when an items characteristics (i.e., discrimination or difficulty) are influenced by a covariate of noninterest (e.g., gender or race). Two types of DIF exist, uniform and nonuniform. The former exists when only an item’s difficulty differs in relation to a nuisance variable, and the latter describes instances where the discrimination (and possibly difficulty) varies in relation to a nuisance variable. The impacts of DIF have previously been explored using simulated data (Roznowski and Reith, 1999; Wells et al., 2002; Li and Zumbo, 2009). These findings indicate that as larger and more frequent instances of DIF arise, an individual’s latent trait estimate becomes more biased, which can have prominent impacts on downstream statistical conclusions such as inflating Type-1 error for group comparisons (Li and Zumbo, 2009). Examples of studies utilizing real data can be found in both education; (Drasgow, 1987) and cognitive data (Roznowski and Reith, 1999; Maller, 2001), across these results are convergent emphasizing how even when bias is not observable based on the number of correct responses biased items may still be present, and these biases make it difficult to compare groups on a theorized unidimensional assessment.

The MIMIC model assesses for DIF by the inclusion of a direct path from the causal variables onto the response patterns of an individual indicator variable (see Figure 1B). By allowing for a direct path between the covariate of interest (i.e., brain volume) and the response patterns (i.e., correctly answering a question) it allows for differences in the item’s characteristics to be modeled after controlling for the latent ability. Through a mediation framework, DIF is present when this direct effect is not fully mediated (Montoya and Jeon, 2020). Another benefit of the mediation framework is that this technique allows the identification of DIF with a reduced number of observations when compared to other DIF identification techniques (Woods and Grimm, 2011; Cheng et al., 2016; Montoya and Jeon, 2020). Finally, the mediation model can be extended to incorporate a moderation to explore for instances of nonuniform DIF (Figure 1C).

To outline the structure of this paper, we explore a simulation study and an empirical study. The simulation study explores differences in estimated causal relationships when using two-stage approaches versus the MIMIC model. The second study is an empirical study with two goals. The first is to identify and explore instances of DIF in relation to neurophysiological data. The second is to illustrate the MIMIC model affords greater sensitivity when trying to identify brain–behavior relationships.

## Simulation study

### Goals

A simulation was performed to explore the amount of bias introduced when defining formative relationships with a two-stage approach. Data were simulated using a MIMIC model drawing on characteristics similar to the empirical example found in this study. The simulated behavioral data were summarized using two methods: a two-parameter IRT and a sum-score based approach. These behavioral proxies were then regressed onto the simulated causal variables also drawn from the same MIMIC model. Differences between the population and estimated relationships are then explored.

### Methods

**Figure 2:**
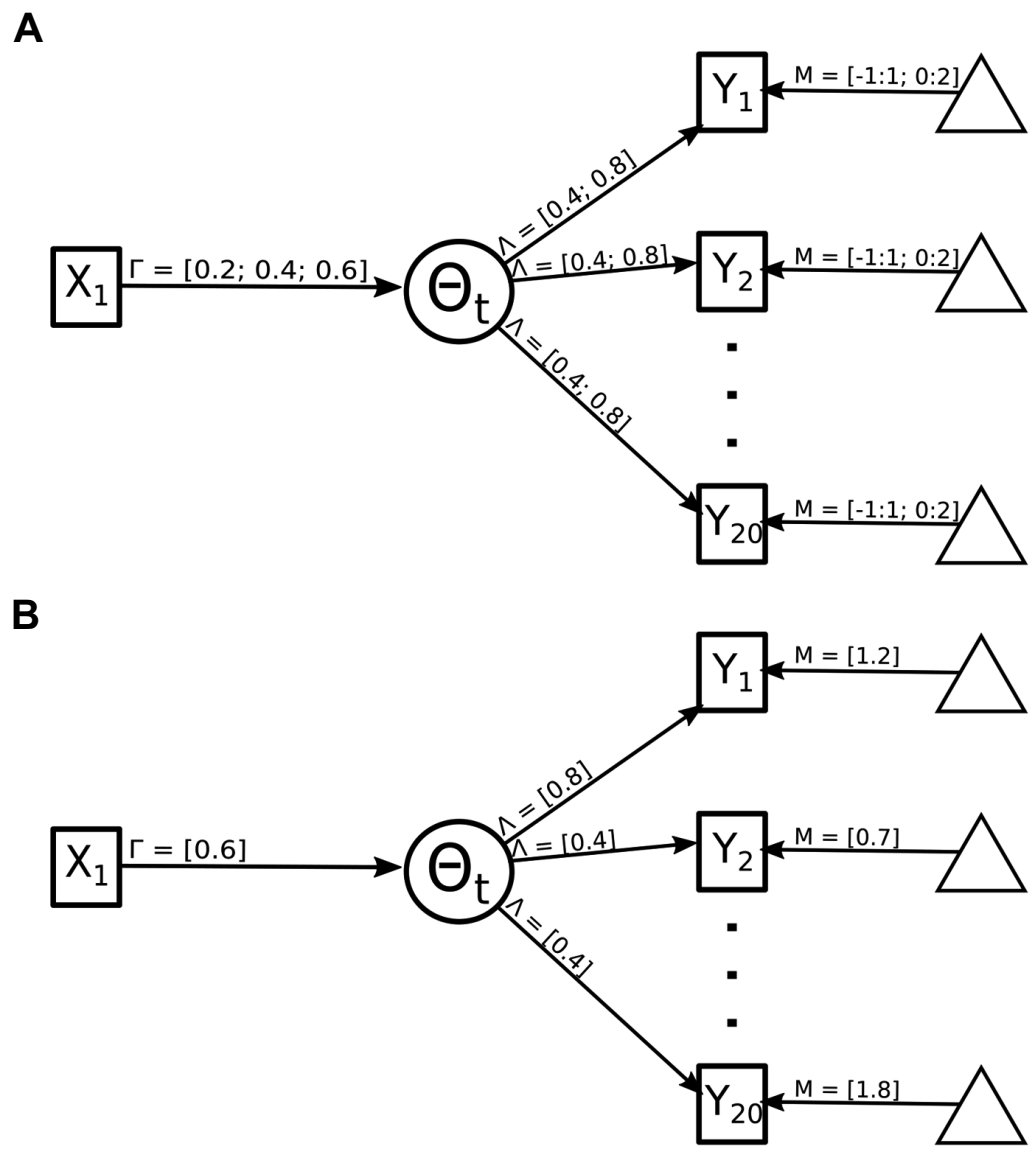
Manipulation of MIMIC model for simulation component. **(A)** Details all possible values that can be sampled from within a single population model. These values include the relationship of the causal model indicated by 𝚪, the strength of the indicator variables indicated by 𝝠, and the intercept values indicated by 𝝡. **(B)** Details one example permutation with 𝚪 = 0.6, the odd 𝝠 = 0.8, the even 𝝠 = 0.4, and the 𝝡 is selected between 0:2 with a uniform distribution.

Simulation conditions were varied in five ways, for a total of 144 various conditions. The conditions included:

1. The number of examinees. This number varied the sample size of the simulated study ranging between a sample size which meets the minimum recommended sample size for an structural equation model exploration (*n* = 200) to a moderately powered exploration (*n* = 500). The minimum recommended sample size follows recommendations from Bollen (1989a) where it is recommended to have about five observations per freely estimated parameter. The moderately powered sample size follows more contemporary recommendations for roughly 10 observations for freely estimated parameters (Christopher Westland, 2010).
2. The strength of the indicator variables. The magnitude of the relationship between the binary indicator variables and the theorized latent variable (i.e., reflexive model) was varied between weak (Beta = 0.4) and strong (Beta = 0.8). The strength of the indicator was selected for the even and odd valued indicators so in total four permutations of the indicator strength were possible (see Figure 2A). This value represents the amount of information an indicator item shares with the latent trait. In an emotional identification setting this can be thought of as a face which is displaying only a single emotion versus traits shared across multiple emotions.
3. Item intercept. This condition type varied the item intercept thresholds—i.e., how high on the latent trait an examinee has to be to have a 50% probability of endorsement. Difficulties of screen items were drawn randomly from a uniform distribution ranging from [−1 to 1] or [0 to 2]. Note that screen item difficulties were never selected from a more difficult range [e.g., (1–3)], because highly difficult screen items inevitably cause such an overwhelming loss of information that the simulations often failed for technical reasons. For example, highly difficult screen items will result in most examinees (rather than only some) endorsing none of the screens and therefore having response vectors of all 0 s (non-endorsements; see Figure 2B). In an emotional identification task this can be extended to how much of an emotion is displayed, anecdotally when an emotion is displayed with greater magnitude, more correct endorsements will be recorded lower the item’s intercept.
4. The magnitude of the causal relationship. The strength of the formative model included values from 0.2, 0.4, and 0.6 (see Figure 2A). The strength of the causal relationship would reflect the true relationship between the theorized brain–behavior relationship.
5. The method used to summarize the indicator variables. Indicator variables were summarized in one of three manners: sum-scores, IRT, and a mimic model. The sum-score approach took the sum of all endorsed items within each simulated participant. The IRT summarized the indicator variables with a unidimensional two-parameter IRT model trained using the “mirt” (Chalmers, 2012) package in R. The last approach used the same approach the data were simulated with, a MIMIC model.

The above five conditions are summarized in Table 1. All simulated conditions used 20 indicator variables, and one causal variable. All permutations were simulated 100 times. All analyses explored parameter bias (True—Estimated) using an ANOVA framework which included all main effects described above and all possible two-, three-, and four-way interactions. Parameter bias was estimated from the sum-score approach by calculating the difference between the population model causal estimate, and the regression weight estimated when the z-scored sum-scores were regressed onto the causal variable. The parameter bias within the IRT framework was estimated by calculating the difference between the population model’s causal magnitude and the regression weight estimated when the ability estimates obtained from a two-parameter IRT model are regressed on the simulated causal variable. Finally, the parameter bias from the MIMIC model is obtained by taking the difference between the magnitude of the population causal relationship with the estimated causal relationship. All simulated datasets were created using MPlus (Muthén and Muthén, 2017), all models used for analysis were trained using R (R Core Team, 2020), all simulation code can be found online.^1^

**Table 1:** Simulation conditions.

Variable	Levels
*n*	200 | 500
Discrimination even	0.4 | 0.8
Discrimination odd	0.4 | 0.8
Magnitude of cause	0.2 | 0.4 | 0.6
Minimum item intercept	−1 | 0

### Results

**Figure 3:**
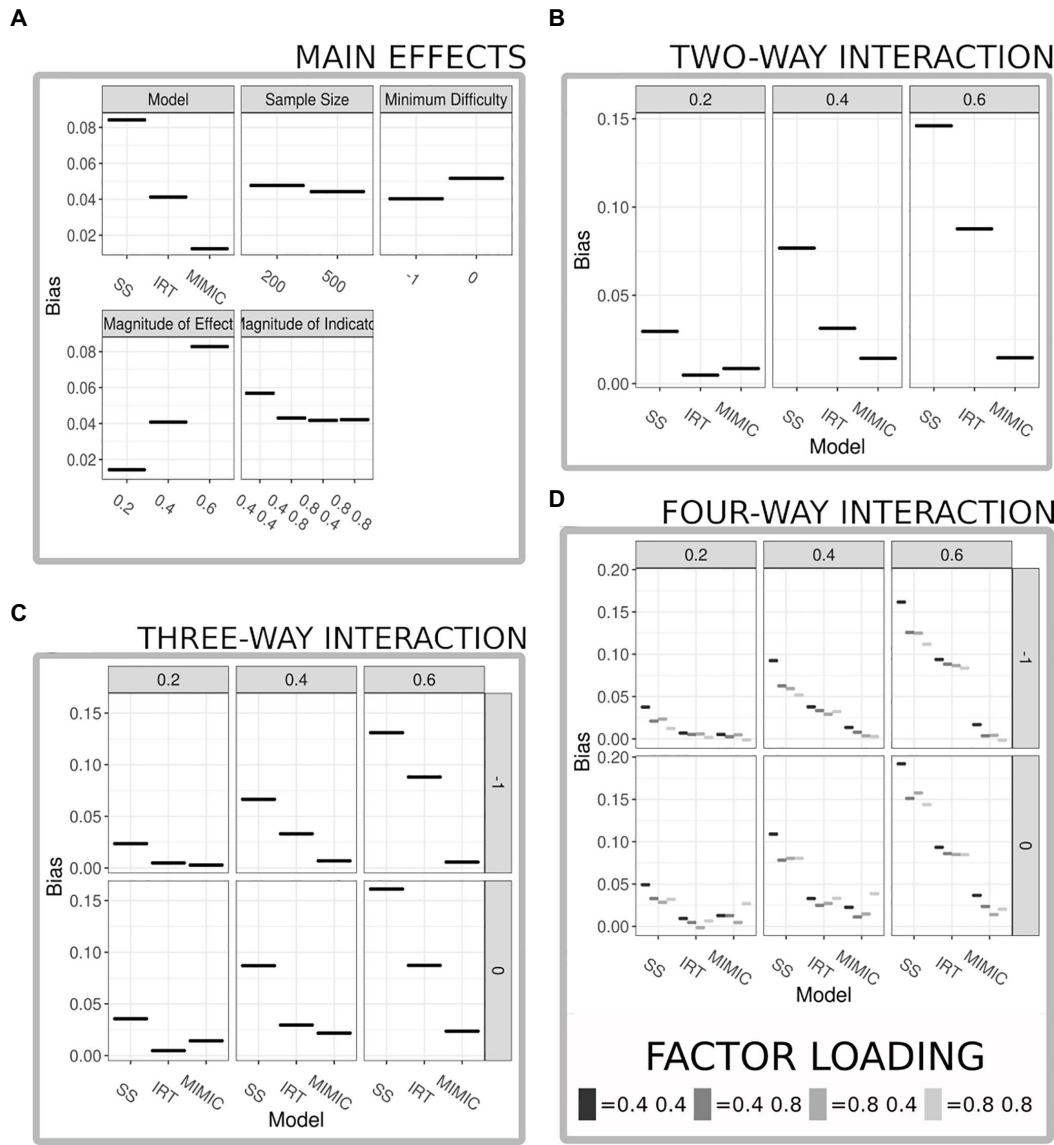
Results from ANOVA comparing bias in parameter estimates. **(A)** Displays the main effects from all variables included in the ANOVA model, panels are faceted by the variable, and the *x*-axis details the levels within each factor. **(B)** Displays the two-way interaction with the largest eta squared between the method used to summarize the behavior scores (model) and the magnitude of the true formative relationship, results suggest near equivalent performance when a weak formative relationship is present across the models, but as the relationship increases the MIMIC model’s bias remains much lower compared to that of the sum-score and item response theory (IRT) model. **(C)** Displays a three-way interaction with the largest eta squared between the methods used to summarize the behavior scores (model) the magnitude of the true formative relationship, and the range of difficulty of the items results extend the logic of the two-way interaction but emphasize the reduction in bias when the difficulty parameters cover a greater majority of the range of ability estimates present in the data. **(D)** Displays a four-way interaction with the largest eta squared between the method used to summarize the behavior scores (model), the magnitude of the true formative relationship, the range of the difficulty parameters, and the magnitude of the indicator variable strength.

Table 2 shows the results of an ANOVA relating the simulation conditions (plus all interactions) to parameter bias. All results are statistically significant, but note that statistical significance is substantially aided by the large number of simulations. Arguably, more meaning can be attached to the ANOVA results by focusing on effect sizes. Table 2 includes eta squared and Cohen’s *F*. Among the main effects, the largest are for the method used to summarize the behavioral data (eta squared = 0.152; see Figure 3A) and the magnitude of the causal relationship (eta squared = 0.142; see Figure 3A). The smallest was for the sample size (eta squared = 0.001; see Figure 3A). The largest two-way interaction was between the method used to summarize the behavioral data and the magnitude of the causal relationship (eta squared = 0.071; see Figure 3B), indicating that all models performed similarly when the causal relationship was weaker, but bias increased much faster for both IRT and sum-scores as the causal relationship strengthened. The strongest three-way interaction extends this pattern to include the item intercept (eta squared = 0.001; see Figure 3C), indicating that bias is lower when items have difficulty values that encompass the majority of the ability distribution (−1:1) as opposed to more restricted difficulty items (0:2). Finally the largest four-way interaction extends the three-way interaction to include the magnitude of the indicator loadings; unsurprisingly, results indicate that a strong indicator set reduces bias across modeling techniques, but this four-way interaction also offers a cautionary note when indicators are weak, sum-scores are used, and the causal relationship is strong, in this permutation the bias was the strongest across all permutations with the estimated effect being on average one-third lower than the population parameter (see Figure 3D).

**Table 2:** ANOVA results predicting by simulation condition.

Parameter	Eta^2^	Cohen’s *F*
Model	0.152	0.424
Magnitude of Cause	0.142	0.406
Model:Magnitude of Cause	0.071	0.276
Magnitude Indicator	0.008	0.091
Item Intercept	0.007	0.082
Model:Magnitude Indicator	0.006	0.077
Model:Item Intercept	0.005	0.068
Magnitude Indicator:Magnitude of Cause	0.002	0.042
Model:Sample Size	0.001	0.038
Magnitude Indicator:Item Intercept	0.001	0.034
Model:Sample Size:Item Intercept	0.001	0.027
Sample Size	0.001	0.025
Item Intercept:Magnitude of Cause	0.001	0.024
Model:Magnitude Indicator:Magnitude of Cause	0.001	0.024
Model:Item Intercept:Magnitude of Cause	0.001	0.023
Magnitude Indicator:Item Intercept:Magnitude of Cause	0	0.022
Sample Size:Item Intercept	0	0.022
Sample Size:Magnitude Indicator:Item Intercept:Magnitude of Cause	0	0.022
Sample Size:Magnitude Indicator:Magnitude of Cause	0	0.021
Model:Magnitude Indicator:Item Intercept	0	0.02
Sample Size:Magnitude Indicator	0	0.019
Model:Magnitude Indicator:Item Intercept:Magnitude of Cause	0	0.014
Model:Sample Size:Magnitude Indicator	0	0.013
Model:Sample Size:Magnitude Indicator:Item Intercept	0	0.013
Sample Size:Magnitude Indicator:Item Intercept	0	0.012
Sample Size:Item Intercept:Magnitude of Cause	0	0.012
Model:Sample Size:Magnitude Indicator:Magnitude of Cause	0	0.012
Model:Sample Size:Magnitude of Cause	0	0.011
Model:Sample Size:Item Intercept:Magnitude of Cause	0	0.008
Sample Size:Magnitude of Cause	0	0.002

## Empirical study

### Goals

The empirical study seeks to underscore how measurement issues and techniques used to describe brain–behavior relationships can alter statistical conclusions. The first portion of the study seeks to explore if DIF can be identified in a behavioral task in relation to neuroimaging data. The second task seeks to identify brain–behavior relationships within this task.

### Methods

#### Approach overview

The goals of this study were two-fold. First, DIF analyses were conducted for a set of emotional identification stimuli through a MIMIC framework. Second, brain–behavior relationships are contrasted across the two-stage approaches and the MIMIC model. These processes required various discrete tasks. First, EEG data were acquired from participants. Second, behavioral data were processed and prepared for an IRT analysis. Third, uniform and nonuniform DIF was assessed using MIMIC models. Fourth and finally, using items that did not display DIF, brain–behavior relationships were drawn between emotional identification and EEG phenotypes using the two separate two-stage approaches and these results are compared against the MIMIC approach.

#### Acquisition of behavioral, demographic, and EEG data

##### EEG behavioral task

Data from 61 participants were acquired for this study. All participants were mothers participating in a larger study on efficacy of home-based interventions for parent–child outcomes, including EEG. Table 3 displays demographic information for all participants. Every participant performed an emotional identification (iDemo) task which included assigning an emotion to a face of a child presented to the participant. The presented faces ranged in one of four possible emotional facial expressions: happy, neutral, discomfort, or distress. Images were presented for 500 ms with a 1,000–1,500 ms inter-trial interval randomized across trials. After faces were presented, participants were instructed to answer which emotion the face displayed. Responses were recorded using a keyboard, using the A, S, K, and L keys. Participants had the entirety of the time between stimuli to select an answer; when multiple response patterns were included for a stimuli, the final selection was included as the answer. There were a total of 24 faces shown within each emotion. The stimuli were counterbalanced for gender (2) and race (4) with 3 of each permutation included. The majority of images were from a validated infant/child database previously used in event-related potential (ERP) emotional processing tasks, with additional images selected from stock imagery to increase racial diversity commensurate with the participant sample (Proverbio et al., 2006, 2007). Additional images were matched in content, style, and luminance to database images. A total of 96 items were included for an entire cycle, participants performed 4 cycles. Every participant had 384 possible responses. Total run time for each task was ~12.5 min. Data were treated as repeated measures, so for a complete battery performance this yielded a data set with four rows and 96 columns of observations.

**Table 3:** Demographic variables for empirical study.

Race	*N*	Age (SD)	Income (SD)	College degree	Vo-Tech School/Training	Some college (no degree)	Grades 9–12 (did not graduate)	High School Diploma or GED	Less than 9th grade
All	61	29.12 (6.57)	19215.22 (22435.71)	0.2	0.12	0.35	0.03	0.28	0.02
American Indian	3	34.15 (4.11)	16,320 (3771.9)	0	0.67	0	0	0.33	0
Black	24	28.1 (6.77)	17325.22 (26660.33)	0.21	0.08	0.42	0.04	0.25	0
Latino	11	27.3 (5.73)	12071.36 (16152.84)	0.09	0.09	0.27	0.09	0.36	0.09
Other	4	22.69 (3.29)	9712.5 (6277.79)	0	0.25	0.5	0	0.25	0
White	19	32.18 (6.08)	29141.65 (21785.58)	0.33	0.06	0.33	0	0.28	0

##### EEG protocol and data processing

Event-related potential measurements were obtained continuously using a 128-channel EGI (Electrical Geodesics, Eugene, Oregon) mobile EEG system, referenced to vertex, filtered 0.01–0.200 Hz, and sampled at 1,000 Hz. Impedances were kept below 50 kOhm. Continuous EEG data collected were filtered 0.5–0.50 Hz and re-referenced to an average reference. Bad channels (maximum 5%) were interpolated using spherical spline interpolation available in BESA software (Brain Electrical Source Analysis, Grafelfing, Germany). Cardiac, eye movement, blink, and muscle-related artifacts were removed using Independent Component Analysis (ICA) in MATLAB (Delorme and Makeig, 2004). Artifact-free trials were then epoched from −250 to 750 ms around each face stimulus, and ERPs were produced by averaging recordings from 26 occipitotemporal electrode sites to best capture the topography of the ERP variable of interest, the P200 ERP. P200 was defined as the largest positive deflection of the averaged waveform between 180 and 250 ms post-stimulus; amplitude and latency was measured at the peak of this deflection. The P200 ERP was chosen as the outcome for this model as it is one of the earliest ERP peaks associated with valenced emotion identification and discrimination (Han et al., 2021), and modulation of the P200 to emotional faces has been associated with emotional regulation skills in adults (Meaux et al., 2014).

#### Preprocessing of behavioral data

Quality assurance on the behavioral data was performed to protect against missingness concerns, or responses that occurred unreasonably quick.

##### Missing responses

Participants who had more than half of the responses missing were marked as outliers.

##### Unreasonable response time

Responses that occurred <150 ms were marked as outliers and coded as NA values.

##### Multiple responses

If a participant provided multiple responses for a question the last reported answer would be selected as the recorded response.

#### DIF identification

**Figure 4:**
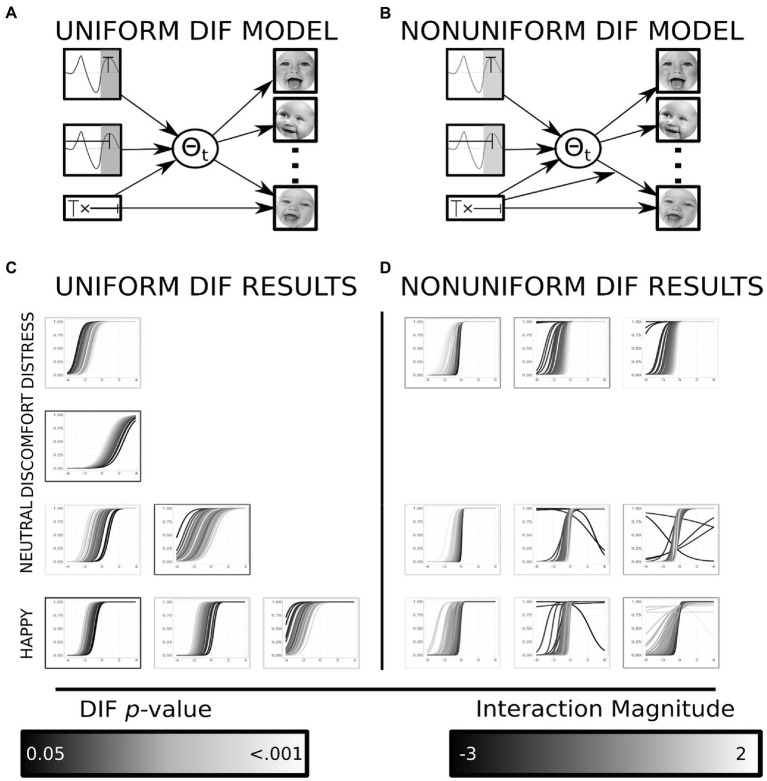
Results from DIF analyses. **(A)** Displays the mediation model used to identify all instances of uniform DIF within an emotion. **(B)** Displays the moderated mediation model used to identify all instances of nonuniform DIF within an emotion. **(C)** The resulting item characteristic curves (ICC) from all uniform DIF items organized within emotion. The color of the ICC is determined by the individual’s interaction (amplitude by latency) magnitude. The color of the border displays the significance of the direct path from the interaction to an item’s response patterns after controlling for the latent variable. The uniform DIF results in changes in difficulty (intercept) displayed by parallel shifts of the item characteristics curve. **(D)** The resulting ICC from all nonuniform DIF items organized within emotion. The color of the ICC is determined by the individual’s interaction (amplitude by latency) magnitude. The border displays the significance of the moderation between the individual’s interaction value and the latent trait onto an item’s response patterns after controlling for the latent variable. The nonuniform DIF analyses explore for changes in discrimination (slope), instances where item characteristics are not parallel indicate nonuniform DIF (Proverbio et al., 2006). Facial images reproduced with permission from Proverbio et al. (2006).

Items that exhibited DIF were identified following previously reported methodology (Montoya and Jeon, 2020). Briefly, this requires a mediation as well as a moderated mediation model to be trained for each item, across all items within an emotion. Models for uniform DIF were tested in a mediation framework using MPlus (Muthén and Muthén, 2017), analytic code can be found online (see Footnote 1). The moderation framework allows for each item’s difficulty parameter to be modeled as a function of the covariates of interest, here the covariates of interest included the P200 latency, amplitude and the interaction of these variables. This brute force DIF analysis follows reported best practice methodology for identifying items that exhibit DIF using a MIMIC model (Wang et al., 2009). When the association between the causal variable (e.g., P200 waveform characteristics, see Figure 4A) and the response for a single item was not fully mediated by the IRT latent factor, this suggested the presence of uniform DIF for the modeled item. The moderated mediation framework allows for the path between the latent variable and the indicator variables (iDemo responses) to vary as a function of the causal variable (P200 waveform characteristics, see Figure 4B). This would suggest the information the indicator variable possesses varies systematically based on an individual’s neurophysiology. The outcome of interest for these models was now the magnitude of the moderation between the latent variable and a specific indicator’s response. Any item which met statistical significance for uniform or nonuniform DIF was removed from any further analyses.

#### Brain-to-behavior relationships

The focus is now on relating iDemo performance to the observed brain phenotypes. This was performed through three alternative techniques which included: sum-scores, IRT, and the MIMIC model. For these analyses brain physiological estimates included the P200 ERP amplitude, latency, and the interaction between the amplitude and the latency. The indicator variables included all questions that did not exhibit any form of DIF. Within each emotion, one model was fitted, across the four fitted models false discovery rate (FDR; Benjamini and Hochberg, 1995) correction was applied across each amplitude, latency, and the interaction, respectively. Any statistical comparison highlighted has been corrected for four alternative comparisons. Due to the nested nature of the data (i.e., multiple behavioral and neuroimaging measurements per individual), standard errors were corrected for possible residual correlations. The MIMIC model corrected for this by estimating the standard errors using the sandwich correction method implemented in MPlus, the two-stage approaches ignored the nested nature of the data to further underscore the increased power that the MIMIC model. In order to compare the behavioral performance of the three techniques, correlations were calculated across all of the three iDemo performance summary values, in order to maintain a similar scale the sum scores were z-scored prior to any comparisons. Next, in order to compare the strength of the relationships drawn across these techniques, the magnitude and significance of the estimated coefficients when the iDemo performance was regressed onto the brain outputs (ERP waveform characteristics) were compared across the three techniques.

### Results

#### Missing data

The mean and SD for trial observations for the iDemo responses and complete EEG time series can be found in Table 4. The lowest average response count for the iDemo task was recorded from the neutral stimuli (mean response count = 21.97) detailing that participants on average had 21 reported responses out of 24 possible presentations. The EEG results suggest the emotion with the lowest average time series recording count were the discomfort stimuli (mean time series count = 23.1).

**Table 4:** Average number of observations per participant per iDemo administration.

Emotion	Average number of iDemo trials	Average number of EEG recordings
Distress	22.48 (3.54)	23.3 (0.20)
Happy	22.99 (2.27)	23.2 (0.20)
Neutral	21.97 (3.92)	23.2 (0.22)
Discomfort	22.09 (3.46)	23.13 (0.24)

#### Uniform DIF

Uniform DIF was tested through a mediation framework. When complete mediation between the causal variable (P200 waveform) and the indicator responses was detected, uniform DIF exists (see Figure 1B). In total seven items displayed uniform DIF, with the results ranging in both magnitude and direction. One distress item displayed uniform DIF; the direction suggested that individuals with a larger interaction between the P200 amplitude and latency term had a lower difficulty than individuals with a lower interaction magnitudes (𝛽_dif_ = 0.504, *t*-statistic = 2.792, value of *p* = 0.005; see Figure 4C). One discomfort item displayed uniform DIF, and the direction of the effect was opposite to that observed in the distress item (𝛽_dif_ = −0.304, *t*-statistic = −2.165, value of *p* = 0.030; see Figure 4C); individuals with lower magnitude interaction terms displayed larger difficulty values than individual’s with greater interaction terms. Two neutral items displayed uniform DIF and were incongruent in the direction of the effect: the first effect suggested individuals with smaller interaction terms had greater difficulty (𝛽_dif_ = −0.712, *t*-statistic = −2.563, value of *p* = 0.010; see Figure 4C), whereas the second suggested the opposite effect (𝛽_dif_ = 0.426, *t*-statistic = 2.089, value of *p* = 0.037; see Figure 4C). Finally, the happy items displayed three instances of uniform DIF. Of the three items, two of these suggested that difficulty estimates were greater in individuals who had lower interaction magnitudes (𝛽_dif_ = −0.503, *t*-statistic = −2.036, value of *p* = 0.042; 𝛽_dif_ = −0.938, *t*-statistic = −3.443, value of *p* = 0.001; see Figure 4), the third item displayed the opposite effect (𝛽_dif_ = 0.511, *t*-statistic = 2.359, value of *p* = 0.018; see Figure 4C).

#### Nonuniform DIF

Nonuniform DIF was tested through a moderated mediation framework assessing if the information an item possesses about a latent trait (discrimination) varies as a function of the causal variables (see Figure 1C). In total nine items displayed nonuniform DIF, and results varied in both direction and magnitude. Three distress items displayed nonuniform DIF; two of the items suggested that the discrimination parameter increased for individuals with greater magnitude of the interaction term (𝛽_dis_ = 0.949, *t*-statistic = 2.440, value of *p* = 0.015; 𝛽_dis_ = 0.738, *t*-statistic = 2.863, value of *p* = 0.004; see Figure 4D); whereas, one item displayed the opposite effect suggesting that as the interaction term increased, the information the item possesses (about the latent factor) decreases (𝛽_dis_ = −0.661, *t*-statistic = −2.271, value of *p* = 0.023; see Figure 4D). Three neutral items displayed nonuniform DIF: two of the items suggested that the discrimination parameter increased for individuals with greater magnitude of the interaction term (𝛽_dis_ = 1.051, *t*-statistic = 2.880, value of *p* = 0.004; 𝛽_dis_ = 0.790, *t*-statistic = 2.400, value of *p* = 0.016; see Figure 4D); whereas, one item displayed the opposite effect suggesting that as the interaction term increased the information the item possesses decreases (𝛽_dis_ = −1.133, *t*-statistic = −2.992, value of *p* = 0.003; see Figure 4D). Three items from the happy paradigm displayed nonuniform DIF, with two of the items suggesting increased discrimination as the magnitude of the interaction term increased (𝛽_dis_ = −0.728, *t*-statistic = −2.829, value of *p* = 0.005; 𝛽_dis_ = −0.504, *t*-statistic = −2.287, value of *p* = 0.022; see Figure 4D); the remaining item showed a positive relationship between the interaction term and the magnitude of the discrimination (𝛽_dis_ = 0.995, *t*-statistic = 3.314, value of *p* = 0.001).

#### Brain and behavior relationships

**Figure 5:**
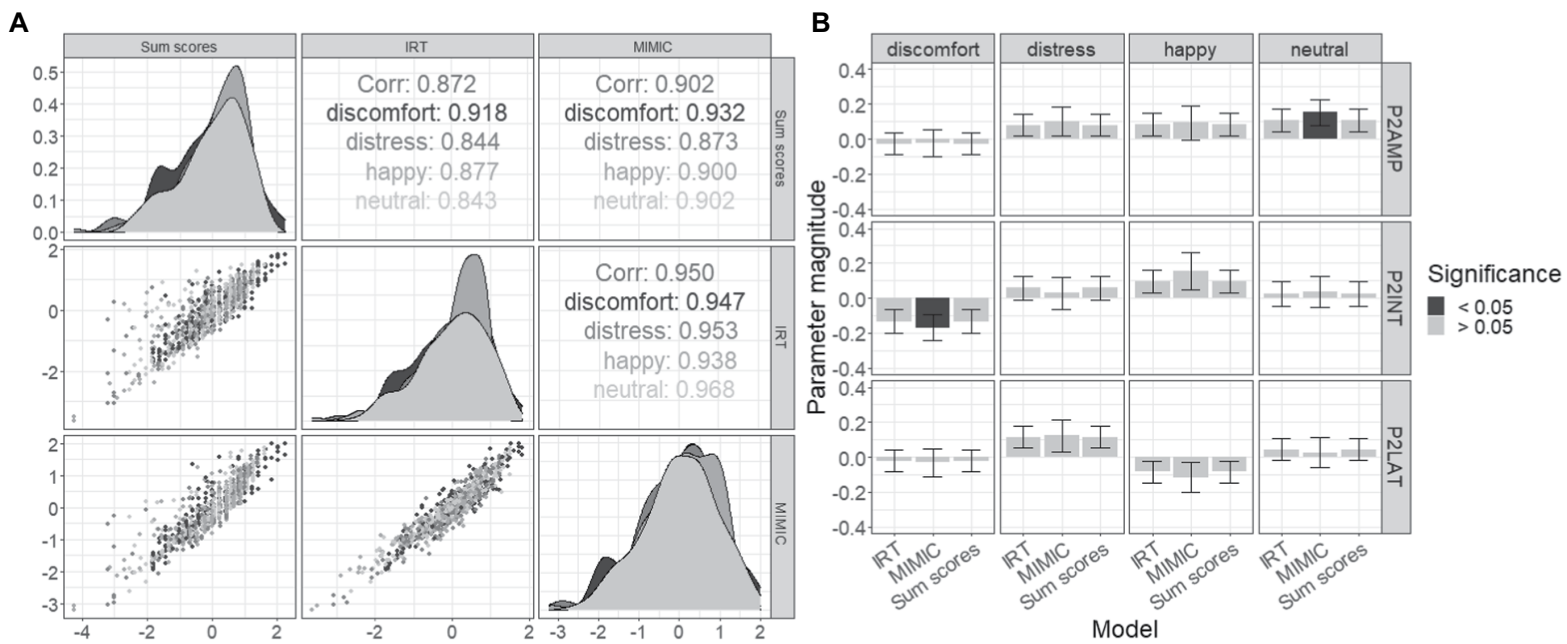
Comparison of MIMIC model and two-stage results. **(A)** Displayed are correlations of iDemo battery summarization which include the two-stage sum-scores, a two-stage IRT, and finally the MIMIC model. **(B)** Magnitude of formative relationships is plotted (+/− SE), with significant effects distinguished from nonsignificant effects based on the bar’s fill.

The final set of analyses sought to compare the separate two-stage approaches with the MIMIC model in both differences across the summary of the behavioral data, and the estimated brain–behavior relationships using the purified itemset. Differences across these techniques in the summary of the iDemo performance are first explored using correlations (see Figure 5). The sum scores displayed the lowest correlation with the IRT approach overall (*r*_overall_ = 0.872) with the minimum correlation being observed in the neutral (*r*_neutral_ = 0.843) and the largest from the discomfort paradigm (*r*_discomfort_ = 0.918). The sum-score approach displayed a greater overall relationship with the MIMIC model (*r*_overall_ = 0.902). Within the emotions, the lowest correlation was observed between the distress performance summary metrics (*r*_distress_ = 0.873), and the largest was again observed in the discomfort paradigm (*r*_unhappy_ = 0.932). Finally, the largest overall relationship was observed between the MIMIC model and the IRT approaches (*r*_overall_ = 0.950). The lowest correlation was observed in the happy paradigm (*r*_happy_ = 0.938), and the largest was observed in the neutral paradigm (*r*_neutral_ = 0.968). All of these reported correlations are significant with value of *p* less than 0.005.

Next, the magnitude and significance of the brain–behavior relationships were explored and compared across all three methods. Two significant effects were observed after FDR correction: the P200 amplitude displayed a positive effect with neutral iDemo performance when estimated within the MIMIC model [𝛽_amp_ = 0.153, *t*(50) = 2.60, *q*-value = 0.04; CFI = 0.907, rmsea = 0.052], and the interaction term displayed a significant negative effect with discomfort iDemo performance when estimated within the MIMIC model [𝛽_int_ = −0.169, *t*(50) = −2.39, *q*-value = 0.02; CFI = 0.952, rmsea = 0.053] furthermore. When these effects were estimated using the two-stage approaches, the direction of the effects agreed but did not display significant relationships at an alpha level of 0.05.

## Discussion

In this paper we present an alternative technique—the MIMIC model—which allows cognitive neuroscientists to fine tune behavioral data toward specific anatomical or physiological neural data. Beginning with a simulation study, the ability to recover theorized formative relationships is compared across the two-stage and MIMIC approaches. Results indicate increased bias in the two-stage approaches, underscoring loss of information when brain and behavior are summarized in isolation. An empirical study was performed to explore two separate issues underlying estimation of brain–behavior relationships: the first is that item sets may show undesirable behavior with respect to an individual’s neurophysiology, and the second illustrates the MIMIC model’s superior performance for the identification of brain–behavior relationships. Through this workflow we have highlighted differences in statistical conclusions when comparing the MIMIC model with two-stage approaches.

### Greater specificity for formative relationships

The MIMIC model is a systems of equations approach which can perform a task similar in nature to that of CCA and PLS, but allows for statistical tests to be performed on both the individual paths within a model, as well as the entire model itself. The benefit of the systems approach is the reduction in bias as highlighted by the simulation component of this study. One of the strongest predictors in the ANOVA was the magnitude of the formative relationship suggesting that as the theorized brain–behavior relationship increases in magnitude, the two-stage approach increases in bias much faster than the MIMIC model. This is important as the range of reported effect sizes within a single modality (volume) predicting general cognition is very large ranging between 3% and greater than 30% of the total variation explained (Gur et al., 2021). Taking the most extreme instances when an R^2^ explains roughly 30% of the variance, which reflects a large effect size in the behavioral sciences, the reliance on typical two-stage approaches may underestimate this already large effect. Taken together the MIMIC model is a versatile modeling technique which is potentially more resilient to intricacies in modeling strong brain–behavior relationships.

### Instances of DIF in relation to neuroimaging data

Across the field of neuroimaging the quality of the physiological and anatomical data has received considerable attention. Approaches for identifying motion and controlling for impacts of motion impacted MRI images exist for anatomical (Rosen et al., 2018), functional (Ciric et al., 2018), and diffusion based analyses (Baum et al., 2018). Similarly, techniques to control for confounding influences of motion exist for EEG data (Liu et al., 2019) as well as ensuring participants are acclimated to the lab testing environment (Brooker et al., 2020). This study highlights how behavioral data can suffer from methodological confounders similar to those found in neuroimaging data. The presence of DIF, with respect to an individual’s physiological characteristics, highlights the importance of assessing the quality of behavioral data across a range of individual characteristics. The motivation for the exploration of DIF is to increase precision of the latent trait estimates (Rupp and Zumbo, 2006). Such studies are influenced by ensuring the dimensionality of the behavioral data is consistent with the models being imposed upon it (Millsap, 2007). That is, when DIF exists, unaccounted for latent variables are influencing the response of an indicator, in relation to an individual’s neuroimaging this suggests the P200 waveform may influence more than a single domain in emotional identification in DIF items. Furthermore, a meta-analysis which explored general cognitive relationships with brain volume concluded that considerable variation in the reported effect sizes can be explained by the quality of the behavioral data (Gignac and Bates, 2017). By performing the DIF analysis in relation to the neuroimaging data, it ensures that the measurement component is tightly coupled to the outcome of interest.

Typically, DIF studies follow a very structured framework where purification of itemsets is attempting to protect against demographic differences. These have historically included variables such as gender, race, or age differences. These demographic variables are typically controlled for in cognitive neuroscience studies, but protecting against these group differences does not ensure the high-quality behavioral data. Few commonly used techniques can be used to identify DIF with respect to continuous covariates (Bauer, 2017). The methodology presented here can incorporate typical nuisance variables, but also ensures the outcome of interest is finely tuned with the independent variable of interest.

### Multiple outcomes for neuroimaging and neurophysiology data

One of the issues of working with neuroimaging data is the proliferation of independent variables. Specific to EEG, and through an ERP framework, a single waveform possesses both latency and amplitude; however, specific to emotional identification and face expression a number of waveforms have been used including the N170, the P200, the P200 and others. Multiple techniques have been applied in order to deal with the number of possible predictors as well as the interrelationships these predictors share. For instance, techniques which have been used to explore functional relationships across neuroimaging and behavioral data include joint ICA (Calhoun et al., 2009), and joint individual and variation explained (Yu et al., 2017) both are examples beyond the already mentioned CCA and PLS. While all of these techniques have their appeals and drawbacks, two major limitations consistent across all of these techniques are the inability to test parametric relationships (path analysis) and the inability to perform model comparisons (Rodgers, 2010). Here, the MIMIC model can satisfy these two limitations, albeit, the MIMIC model requires a more theory driven perspective applied to the data then techniques such as CCA. Through the currently presented ERP framework these techniques can be used within the typical EEG analytic workflow. We have highlighted here how even within a single calculated ERP waveform multiple outcomes can be used. The utilization of SEM has seen some considerable interest when working with the high dimensional data that are the hallmarks of neuroimaging studies. For instance, Bolt et al., addressed the limitations of a region-of-interest based approach by incorporating the hierarchical nature of the brain into a SEM based approach (Bolt et al., 2018). This approach is flexible to the number of ROI’s possible, it can account for interrelations across these regions and, most importantly, allows for the estimation of brain–behavior relationships within a single model. Similar approaches have been pursued using EEG data and behavioral data (Grandy et al., 2013).

### Improved statistical power of MIMIC model in relation to two-stage approach

One of the major highlights from the analyses presented in this study is the strength of the relationship drawn between brain and behavior in the MIMIC model when compared to the two-stage approaches. Emotion identification is a field of study which has a strong literature backing the neural underpinnings of performance in these tasks. Relationships have been studied using EEG data (Bentin and Deouell, 2000; Schupp et al., 2006; Curtis and Cicchetti, 2011; Nemrodov et al., 2018) and functional magnetic resonance imaging (Gur et al., 2002), all of which are supported by behavioral explorations (Ekman, 1992; Erwin et al., 1992; Indersmitten and Gur, 2003; Ciarrochi et al., 2008). This study displays relationships between the P200 waveform and emotional identification capabilities. The results were specific to the lower intensity emotions (i.e., neutral and discomfort) and specific to various characteristics of the waveform such as the amplitude for neutral faces and the interaction of amplitude and latency for discomfort faces. The P200 amplitude showed a positive relationship with emotional identification capabilities for neutral faces suggesting that larger P200 waveforms relate to better identification performances. This is in line with previous reports detailing improved attention to emotional stimuli as the magnitude of the P200 waveform increases (Schupp et al., 2006). The second significant finding details an interaction between the P200 amplitude and latency and how smaller interaction values relate to improved identification capabilities for the discomfort paradigm. Smaller, or negative interaction terms are produced by either a large magnitude and short latency or a small magnitude and long latency waveforms. The interaction between amplitude and latency is not regularly explored in ERP analyses, albeit distinctions between processing time and amplitude do receive attention across emotional paradigms. One example includes distinctions between angry and happy faces where anger receives quicker and smaller P200 characteristics when compared with happy stimuli (Ding et al., 2017). The discomfort paradigm reflects a less intense negative emotion, however, composites of prototypical anger identification yielded relationships with improved identification. That is, while angry faces receive short time to peak P200, this reflected one mechanism for successful identification for discomfort faces when paired with large amplitudes (relative to the mean of this sample); the alternative (long latency, low amplitude) reflects a relatively novel finding for EEG literature in terms of successful emotion identification.

Given the underlying theory, it is worth noting the lack of nominal significance from the two-stage approaches in a dataset which violates the assumptions of linear regression (correlated errors). Even with this error which inflates Type-1 error, the model still fails to identify a significant effect in what is a theoretically motivated relationship. Much like CCA, the MIMIC model finds the linear combinations which maximize the relationships between the manifest variable and the estimated latent trait; this increase in magnitude of estimation is displayed by the significant (*q* < 0.05) effects. In order to further distinguish this benefit of the systems of equations, compare the component solutions derived from a PCA and those derived from a CCA. The estimation of the CCA solution requires that the correlations between the individual component solutions be maximized in their estimation. Accordingly, the correlations across components will be greater in the CCA framework when compared with the PCA framework. The formulation of the MIMIC model follows a similar framework where the relationship between the latent variable and the causal variables is maximized. The directions of the effect derived from the MIMIC model and the two-stage approach all agree. The major appeal comes from a reduction in the standard error, and an increase in the parameter magnitude which leads to two statistically significant effects using the MIMIC model after false discovery rate correction. Furthermore, the MIMIC model allows for measurement error to be removed from the variance of the latent trait allowing for the parameters to be constrained within somesense of the true variance. Finally, it is worth noting that across the three approaches, the parameter directions all agreed with one another, further underscoring that effects were similar but statistical power is inflated through the MIMIC approach.

## Limitations section

The limitations of the simulation study were the relatively narrow parameters used to simulate data as well as the mechanism used to simulate the data. The parameters and effect sizes sampled were drawn from the empirical portion of this study with specific focus on emotional identification. The second limitation includes the single method used to simulate data: the MIMIC model, future studies should explore alternative techniques to simulate data.

Limitations of the empirical study include a limited sample size with repeated measures. However, the intraindividual variability of the behavioral responses remained low, whereas variability across individuals remained high. The participant sample also derived from a population of parents undergoing home-based parenting interventions, which may limit generalizability of the specific brain–behavior relationships described here. The home-visit nature of the EEG acquisition also required more aggressive preprocessing techniques. The number of dependent variables was also limited to outcomes suggested by the literature to be of greatest relation to the performance on emotional face identification across valence and intensity (Meaux et al., 2014; Han et al., 2021); while the MIMIC model can incorporate a larger number of causal variables, the selected variables were limited to best compare the performance of regression (across all 3 summary measurement approaches) with respect to a set of theoretically validated ERP components. Finally, all approaches were reliant upon the null hypothesis significance test which assesses if the parameters were different from zero, future researchers should apply a more theory driven assessment of models comparing model parameters estimates with models presented within the field.

## Conclusion

This study sought to display the utility of the MIMIC model for cognitive neuroscientists. The simulation component underscores how the formative relationships are best captured in a systems of equations approach when compared to a two-stage approach. An empirical study was presented to underscore two benefits of the MIMIC model: the first is the ability to explore for DIF in itemsets and the second displays superior sensitivity to theorized brain–behavior relationships. The former point is important as the quality and consistency of cognitive data do not receive the same amount of attention as does neuroimaging data in the typical workflow limiting the parsimony of results. The latter point highlights the increased sensitivity of the MIMIC model to identify brain–behavior relationships even when working with a limited sample size. Moving forward it is the authors’ recommendation that the MIMIC model is used to ensure the greatest quality of behavioral data and the largest brain–behavior relationships are acquired within cognitive neuroscience explorations.

## Data availability statement

The raw data supporting the conclusions of this article will be made available by the authors, without undue reservation.

## Ethics statement

The studies involving human participants were reviewed and approved by the University of Oklahoma Health Science Center Institutional Review Board. The patients/participants provided their written informed consent to participate in this study.

## Author contributions

AR aided in study design, analyzed data, and wrote manuscript. EA analyzed data. NW acquired data. AP provided behavioral stimuli. HS aided in design and analyses. LE oversaw acquisition and processing of neuroimaging data. DB oversaw study design, data analyses, and manuscript preparation. All authors contributed to the article and approved the submitted version.

## Funding

Support of this work was partly funded by the Maternal, Infant, and Early Childhood Home Visiting Grant Program by the Health Resources and Services Administration (grant numbers: UH4MC30745 and D89MC28275).

## Conflict of interest

The authors declare that the research was conducted in the absence of any commercial or financial relationships that could be construed as a potential conflict of interest.

## Publisher’s note

All claims expressed in this article are solely those of the authors and do not necessarily represent those of their affiliated organizations, or those of the publisher, the editors and the reviewers. Any product that may be evaluated in this article, or claim that may be made by its manufacturer, is not guaranteed or endorsed by the publisher.

## Footnotes

^1^ https://github.com/adrose/mimicDifEEGAnalyses
